# Intra- and Interexaminer Measurement Variability Analysis of an Orthodontic Gauge Device to Determine Incisor Occlusal Surface Angles in the Horse

**DOI:** 10.3390/vetsci9090481

**Published:** 2022-09-07

**Authors:** Silvio Kau, Katharina S. Motter, Viktoria J. Moser, João R. Kunz, Matteo Pellachin, Bettina Hartl

**Affiliations:** 1 Institute of Morphology, Department of Pathobiology, University of Veterinary Medicine Vienna, Veterinärplatz 1, 1210 Vienna, Austria; 2 Department of Equine Dentistry, Maxillofacial Diagnostic Imaging and Surgery, Veterinary Clinic Gessertshausen Altano GmbH, Grasweg 2, 86459 Gessertshausen, Germany; 3 Private Equine Veterinary Dentist and Specialist on Equine Orthodontics, Via Marino Grimani 7, 35127 Padova, Italy

**Keywords:** equine, tooth angle, occlusal surface, incisor table angle, malocclusion, diagonal bite, angle measurement, measuring accuracy

## Abstract

**Simple Summary:**

Annual tooth rasping is integral to prophylaxis work in equine dentistry. The procedure begins with a thorough examination of the oral cavity, including assessment of incisor occlusal surface angles. Any dental misalignment should be corrected or reduced to prevent abnormal forces from acting on teeth and supporting tissues and to maximize functionality of the masticatory apparatus. Thus far, clinical methods for dental angle assessment are scarce and not validated in terms of data reproducibility and comparability. These parameters are vital to objectively assess treatment needs and outcome, obtain population data, and establish data comparability among studies. However, this is the first study systematically validating a commercial dental angle measuring device. The data indicate high device performance and its applicability in practice.

**Abstract:**

Incisor malocclusions are common in horses. As yet, an evidence-based understanding of incisor occlusal surface angle dynamics and normocclusal range is missing. Orthodontic measuring devices could help unravel this information objectively but imply measurement validation. We evaluated intra- and interexaminer variability of repeated sagittal and transversal incisor occlusal surface angle measures using a commercial orthodontic gauge device (*MaPHorse1*). Five examiners (two experienced, three inexperienced) measured six cadaver heads on 2 consecutive days in a blinded block-randomization design, resulting in 16 measures per examiner*head. Sagittal and transversal angle measures revealed low intraexaminer variability at scale-level independent mean SDs of α 0.58° and α 0.69°, respectively. Sagittal angle measures associate with low interexaminer variability, showing small mean angle differences (max. α 0.51° ± 0.35°), small scatter, and more consistent data reproducibility. Despite comparable mean interexaminer differences, the spread of transversal angle measures was relevantly higher using the proposed landmarks (average 2.2-fold higher interquartile range). The measurement performance of experienced and inexperienced examiners did not systematically differ. The time required for individual measurements was already comparable after 24/96 repetitions. This instrument may help deciphering sagittal angle normocclusal range and orthognathic dynamics and, with a proposed procedural amendment, transversal angle as well.

## 1. Introduction

Occlusal surface angles in equine pre- and post-canine dentition receive increasing attention as a certain range of inclination is accepted as crucial for physiological mastication and, thus, feed conversion [1,2,3,4]. It was shown that the extent of occlusal angulation in equine cheek teeth depends on mandibular deflection during chewing [5], which, in turn, is related to forage particle size [6]. However, there is no evidence-based consensus of what influences incisor occlusal surface angle dynamics and defines a normal healthy inclination range or pathological deviations from it. Some authors assume that inclination of incisor occlusal surface angles remains constant with increasing age [2,7,8,9,10], although equine incisors are subjected to distinct age-related morphometric changes. Studies have shown a decrease in tooth length due to progressing apexification and resulting wear and eruption imbalance [11,12], tooth position-dependent decrease in interincisal angulation [13], and resulting changes in geometry and appearance of the occlusal surface [12,14].

**Figure 1:**
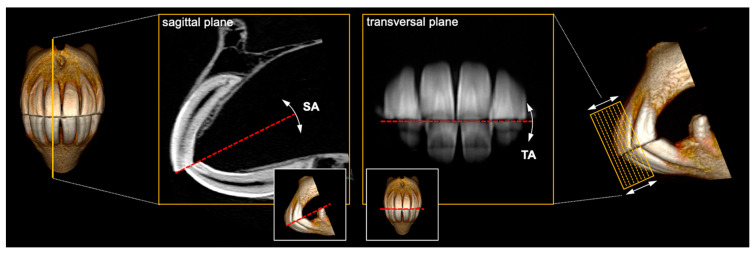
Exemplary depiction of sagittal angle (SA) and transversal angle (TA) constituting the two-dimensionally inclined incisor occlusal surface. SA inclination is shown on a 0.6mm CT section of teeth 101 and 401; TA inclination is shown on a mean intensity projection (MeanIP) thick-slab reconstruction of series of such CT sections, including clinical crown parts of all Triadan 01 and 02.

Incisors can be overlong, which is favored by less abrasive forage and resulting lack of wear and can reduce functional cheek tooth occlusion [4,15]. Therefore, incisor shortening is accepted as state of the art in routine odontoplasty in horses [4,16] but requires careful case-specific pre-treatment assessment [8,17]. Consideration of individual occlusal surface angles of incisors is imperative to any corrective procedure changing incisor length or occlusal surface structure. Orthodontic measuring devices can be a helpful tool, but there are currently no validation studies on reported measuring methods. In general, incisors are assessed before the mouth gag is inserted [18]. To determine the two-dimensional inclination, occlusal surface angles are assessed both from the side and the front [19,20]. The angle determined from the side, viewing at the incisor profile, was recently defined as the sagittal angle (SA), whereas the one determined from the front is referred to as the transversal angle (TA) (Figure 1) [2].

SA indicates the rostro-caudal inclination of the occlusal surface in reference to a global or subordinate transversal plane [2,3,20]. The terms “incisor table angle” [21,22] and “temporomandibular joint angle” [20] are also used for SA. Elsewhere, however, the term “angle of the incisor occlusal table” was used for TA [4]. Other authors refer to the angulation of the incisor occlusal surface as “occlusal angle” [23,24]. Dixon (2002) also described the “occlusal angle” as the angle encompassed by incisor clinical crowns in a profile view [25]. We refer to Listmann et al. (2017), who defined the overall inclination of the incisor’s occlusal surface as the incisor table angle and emphasized its two-dimensionality by introducing the terms SA and TA [2]. Described landmarks and proposed physiological values vary substantially for SA. Studies reported that the extended occlusal surface plane should cross the eye or ear ground [9,10], the temporomandibular joint (TMJ) [26], should be α 5° above the TMJ [20], and should be parallel to the facial crest [8] or α 10–15° to the mandibular interalveolar margin [7]. Others measured the mean SAs of α 32.7° (MIN) and α 44.9° (MAX) to a transverse reference plane but avoided referring to values as physiological due to small sample size [2]. Computed tomography-based single tooth analyses revealed SAs to exhibit slight intraindividual intra-arcade asymmetries and marked differences in upper and lower jaw incisors, the latter exhibiting up to 30% steeper angles [2].

TA indicates the latero-lateral inclination of the occlusal surface in reference to a global or subordinate sagittal plane [2,3,20]. Described landmarks and proposed physiological values are rather uniform for TA. In centric occlusion, the opposing incisors close almost isognathic to each other [18] and show a level incisor bite when viewed from the labial side [27,28]. Some authors assume that the physiological transverse angle is perpendicular to the median/sagittal plane [2,7,8,19] and, accordingly, that upper and lower jaw incisor arcades meet in a plane that is parallel to the ground [4,19]. In reference to a sagittal plane, TAs were recently measured, ranging from α 0° to 22.5° with mean values of α 3.5°–6.8° [2]. Deviation in TA from assumed normocclusion is known as diagonal incisor malocclusion (DIM) [29]. In DIM, diagonally positioned incisors are too long, resulting in a tilted occlusal surface inclination [4]. A DIM is termed DGL-3 when quadrants 100 and 300 are overgrown, or DGL-4 when quadrants 200 and 400 are overgrown [20].

Malocclusions can impair dental and periodontal function and require individualized treatment to maximize functional occlusion [23,30,31]. Currently, most equine veterinarians assess occlusion, or, rather, malocclusion, subjectively by adspection or using unvalidated measuring devices. Some use a metric ruler to obtain the lateral jaw excursion to molar contact (EMC) [4,8]. Pimentel and Zoppa (2014) inserted a Bussico gauge between incisors to evaluate their occlusal alignment [17]. Allen (2008) determined the angle between incisor occlusal surface planes and mandibular interalveolar margins using a protractor [7]. Except Kunz et al. (2020), no other peer-reviewed study has used *MaPHorse1*. However, in their research, the orthodontic gauge device was utilized for sole determination of DIM direction, i.e., DGL-3/-4. According to the authors, no metric angle values were reported as measurement repeatability and reproducibility had not yet been investigated for the device [22].

This cadaveric study aims to assess intraexaminer variability (repeatability) and interexaminer variability (reproducibility) of both SA and TA measurements using the orthodontic gauge device *MaPHorse1*. The study was conducted following a blinded two-day block-randomization design where five examiners of different experience status repeatedly measured SA and TA on six cadaver heads.

## 2. Materials and Methods

### 2.1. Cadaver Heads

Six fresh-frozen (*n* = 5) or fresh (*n* = 1) heads of horses from the anatomical collection of the University of Veterinary Medicine Vienna were used. Horses were euthanized at the Equine Clinic of the university for reasons unrelated to the study or any orodental, maxillofacial, or cranial disease. Heads were resected at the level of the atlas. Details on the animals and cadaver heads are listed in Table 1. The frozen heads were stored at −18 °C and thawed over 2 days in a water bath at 4 °C before the experiment started. Inclusion criteria for the cadaver heads were a complete number of incisor teeth, permanent dentition, integrity of coat and skin, and the presence of both eyes. Before the measurements, small hooks at the distal border of corner incisors 103 and 203 of horse 3 and 5 were removed using a power float handpiece (H.28, Foredom Electric Co., Bethel, CT, USA).

The experimental set-up corresponds to the guidelines specified by the local ethics committee of the University of Veterinary Medicine Vienna (Austria). According to the study design, no ethical approval for animal experimentation was required.

### 2.2. Examiners

Five examiners performed the measurements (B.H., K.S.M., V.J.M., J.R.K., M.P.). Two of them (J.R.K., M.P.) already had extensive experience with the measuring device over a period of several years before the trial started. Three examiners (B.H., K.S.M., V.J.M.) had no experience using the instrument at the beginning of the experiment.

### 2.3. Orthodontic Gauge Device

We used the commercially available orthodontic gauge device *MaPHorse1* that was designed for clinical determination of equine incisor occlusal surface angles, i.e., SA and TA. The instrument was designed and developed by one of the coauthors (M.P.) and patented by the United States Patent and Trademark Office under patent no.: US 8,793,888 B2 [32].

**Figure 2:**
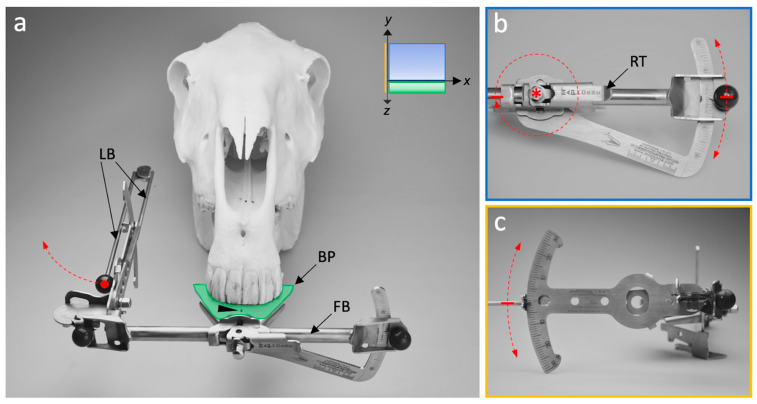
Main mechanical components of the *MaPHorse1*. (**a**) Dorso-rostral view; the bite plate (BP) is positioned between incisor arcades. The central marking line (arrowhead) is aligned to the interdental space of central incisors 101 and 201. The lateral bar (LB) can be folded. FB, frontal bar. (**b**) Frontal view of the TA protractor. Retainer (RT) preventing free FB movement around a rotational connection (asterisk) with the BP; induced movement is possible. FB moves in a transversal plane (*xy*). (**c**) Lateral view of the SA protractor. LB moves in a sagittal plane (*yz*).

The device consists of two elongated interconnected bars, i.e., lateral bar and frontal bar (Figure 2a), each connected to a protractor (Figure 2b,c). Protractors are angled orthogonally to each other. The frontal bar is flexibly connected to a horizontal bite plate and rotatable (α 180°) around a rostro-caudal axis after unfastening a central retainer (Figure 2b). The lateral protractor is parallel to the median/sagittal plane, translating it outside the head. Considering predefined anatomical landmarks, the lateral bar moves in a sagittal plane and determines the SA. The frontal bar moves in a transversal plane and determines the TA. After α 180° rotation of the frontal bar, SA can be measured on the other side as well. The protractor scales exhibit integer angle increments (α 1°).

### 2.4. Experimental Set-Up

The experiment was carried out on two consecutive days in the dissection room of the Institute of Morphology of the University of Veterinary Medicine Vienna. One of the authors (S.K.), who did not perform measurements, organized preparation and coordination of the experiment.

**Figure 3:**
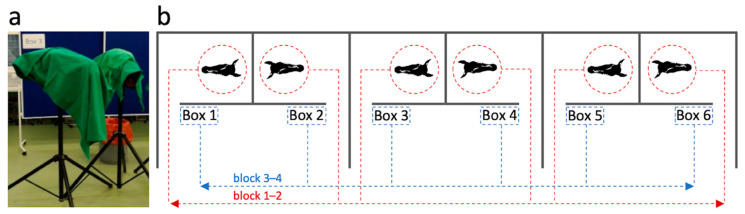
Depiction of the experimental setup. (**a**) Heads fixed on head rests. (**b**) Plan view of examination box setup and head-to-box assignment scheme.

Heads were mounted on custom-made headrests (Figure 3a) 12 h before the trial started and stored at 4 °C in a cold storage room. Mounting was performed using 4-point-fixation with tapered cross locking bolts, i.e., rostrally at the mandibular corpus and caudally at the mandibular ramus. Heads were fixed in a position where measurements would also be taken in live horses. To hold the bite plate between upper and lower incisors after insertion, an elastic stocking was tied snuggly around the nasal dorsum and the rostral mandible. Heads stayed fixed with tapered bolts on the headrests throughout the experiment and were always covered with a cloth outside the examination boxes and between measurements. Heads were stored at 4 °C between the experiments of day 1 and 2. The measurements took about 6 h each day. During measurements, the heads were separately placed in boxes that were separated by view screens (Figure 3). On each day, 2 measurement blocks were conducted. Each block consisted of 4 passages. All examiners measured each head once per passage. Heads were randomly assigned to the boxes after each passage following a block-randomization list (Table S1). The block-randomization list was generated by means of a random number generator for the fixed factors block, passage, and box in the random range 1–6 using Microsoft Excel (version 2016, Microsoft Corp., Redmond, WA, USA). Either the heads or box labels rotated (Figure 3b).

In each passage, the examiners moved from box to box following a separate examination protocol (Table S2). The protocol was exchanged after each measurement block. Obtained values from each passage were hidden so that subsequent measurements were not influenced.

### 2.5. Angle Measurements

**Figure 4:**
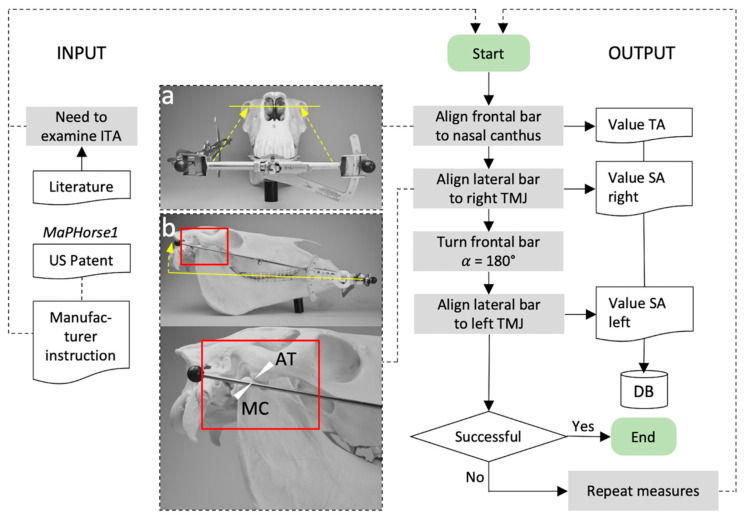
Flow chart of incisor table angle measures using *MaPHorse1* in this validation study. (**a**) Frontal bar alignment to measure the transversal angle (TA). (**b**) Lateral bar alignment to measure the sagittal angle (SA). The lateral bar is centered between the *Tuberculum articulare* of the *Processus zygomaticus ossis temporalis* = articular tubercle (AT) and *Caput mandibulae* of the *Processus condylaris mandibulae* = mandibular condyle (MC) after transcutaneous palpation. ITA, incisor table angle; DB, database.

A total of 3 measuring devices were used parallel in the data collection. This allowed 3 examiners to take measurements at the same time. Before the experiment started, all devices were calibrated by the manufacturer according to a standard procedure. One of the experienced examiners (M.P.) demonstrated the handling of the device and landmarks to be used on a separated cadaver head before the experiment started. Figure 4 merges all steps that were taken for measuring TA and SA (right, left). The procedure is identical as would be performed in live horses. A full dynamic 3D reconstruction of the TA and right-sided SA measuring procedure is shown in video S1.

The bite plate was inserted between incisor arcades until the ventral stop ridge (lower side of bite plate) touched the lower central incisors. The central marking line of the bite plate was aligned to the median interdental space. Then, the frontal bar was positioned in plane with a line connecting the medial canthi of the eyes and the TA was read off the measuring scale. The measurement was performed sitting on a height adjustable seat. The SA was first measured on the right and then on the left side. The unfolded side bar was adjusted to the most lateral palpable part of the TMJ gap. For measuring the SA on the left side, the central retainer was loosened and the frontal bar turned α 180° so that the side bar was positioned on the left side. The retainer was closed and the previously measured TA value was reset on the frontal angle scale. Then, the left SA was assessed as described for the right side. Measured values were immediately recorded in the examination protocol. After the left SA measure, the device was removed from the head and the examiner went to the next box, continuing measurements in the respective passage. Non-measuring examiners had to wait off the experimental setup.

### 2.6. Time Measurements

To identify time budget differences between experienced and inexperienced examiners and to investigate whether repeated use of the measuring device has a quickly recognizable handling learning effect, the time required for the measuring process was assessed for each examiner in different measurement blocks. Time measurement began with the insertion of the biteplate between the incisors and ended with the removal of the device.

### 2.7. Statistical Analysis

Analyses and data visualization were carried out using GraphPad Prism (GraphPad Software Inc., San Diego, CA, USA) and Excel (Microsoft Corp., Redmond, WA, USA) software. All data were analyzed using descriptive statistics and checked for normal distribution using Shapiro–Wilk test. Homogeneity of variance was determined using Levene’s test. Intraexaminer variability (repeatability) was determined using coefficient of variation (COV) calculations as ((SD/mean)*100). The respective scale level of angle measures was considered for interpretation of COVs. Used inferential statistical methods are shown in the respective results section.

## 3. Results

We used a blinded block-randomization study design to evaluate intra- and interexaminer variability for repeated SA and TA measures using orthodontic gauge device *MaPHorse1*. Data collection was performed on 2 consecutive days, each divided into two measurement blocks and four respective measurement passages. Each head was measured once per passage, resulting in a total of 192 SA (right, left) and 96 TA measures per examiner and 32 SA (right, left) and 16 TA measures per examiner*head. Thus, all examiners collected 960 SA (right, left) and 480 TA values. Examiners 1 and 2 entered passage values for heads 1, 3, and 5 (ex1), and head 4 (ex2) in the list once, illegibly. These data clusters were completely removed from the analyses, resulting in 30 SA (right, left) and 15 TA measures per examiner*head.

### 3.1. Landmark Identification Proved Difficult with TA but Not SA Measurements

Five frozen-thawed heads (heads 1–5) and one fresh head (head 6) were available for study purposes. Identification of bony landmarks to measure SAs was reported as easy by all examiners. As by evaluation of the study coordinator (S.K.), no changes in the cadaver material were observed in the TMJ regions over the period of both study days. The bulbi of previously frozen heads showed slightly progressive enophthalmos and distortion of the lateral canthi. The medial canthi remained largely unaltered. However, all the examiners individually reported difficulties in identifying the latter for TA measures. This was not reported for the fresh head, in which anatomical structures appeared unaltered. Both experienced investigators quoted that frozen-thawed specimens were not ideal for measuring the TA. Experienced examiners further stated that this was not comparable to live horses. No difference to live horses was reported for SA measures or the fresh head in general.

### 3.2. SA Measures Revealed Negligible Side Difference within All Examiners

**Figure 5:**
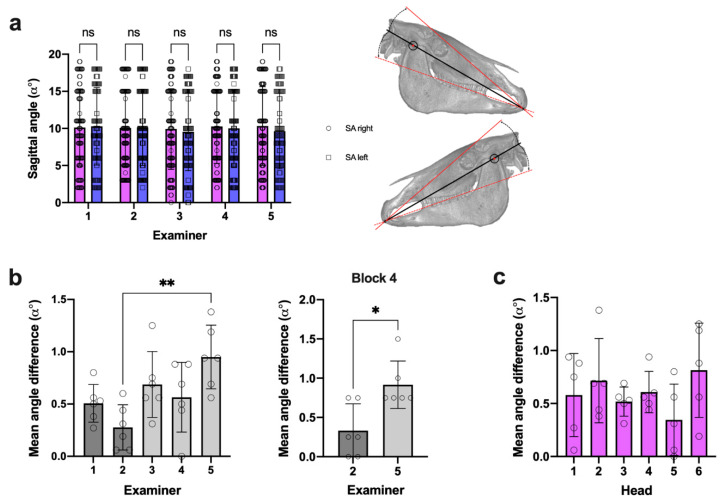
SA side difference comparison. (**a**) All measures per side*examiner; right and left *n* = 93 (ex1), *n* = 95 (ex2), *n* = 96 (ex3–5); two-way ANOVA, *p* = 0.523. (**b**) Mean SA side difference per examiner (dots = mean for individual heads); *n* = as in (a); one-way ANOVA, *p* = 0.005; Tukey’s multiple comparisons, ** *p* = 0.003; Mann–Whitney U test, * *p* = 0.033. (**c**) Mean SA side difference per head (dots = mean for individual examiner); *n* = as in (**a**); one-way ANOVA, *p* = 0.359.

The SA was recorded twice for each measurement using the same anatomical reference structures on the right and left side of the head. The obtained values indicate no marked difference of right and left SA measures among all the examiners (Figure 5a; Table S3). In 52% of all the measures, there was no angle difference at all. Thus, right and left SA values were averaged for intra- and interexaminer variability analyses.

Mean right vs. left SA difference, integrating measures of all heads and examination blocks, was below α 1° and only differed between examiner 2 (experienced) and examiner 5 (inexperienced) (Figure 5b). This was also observed in block 4 (where inexperienced examiners already performed 144 SA measures), but to a lesser extent (Figure 5b). The observed differences were comparable for all heads (Figure 5c).

### 3.3. Intraexaminer Data Repeatability Was Comparable in SA and TA Measures

We determined the coefficient of variation (COV) for each examiner and head as a measure of intraexaminer variability (repeatability) of SA and TA measurements using *MaPHorse1*. In a total of 476 repeated TA measures, 1.1% (*n* = 5) revealed a DGL-4, 88.2% (*n* = 420) a DGL-3, and 10.7% (*n* = 51) no TA deviation (TA α 0°) (Table S4). Due to mathematical reasons for COV calculations, negative TA values (DGL-4) and cases with a SD > mean (mean values near α 0°) were excluded. This applied for examiner*head combinations (mean TA ± SD) 2*1 (−0.06° ± 0.25°), 4*1 (−0.06° ± 1.06°) and 5*1 (0.25° ± 0.86°). Therefore, a total of 3x16 values were dropped from the COV calculations despite exhibiting absolute dispersion of measurements comparable to other examiner*head combinations.

**Figure 6:**
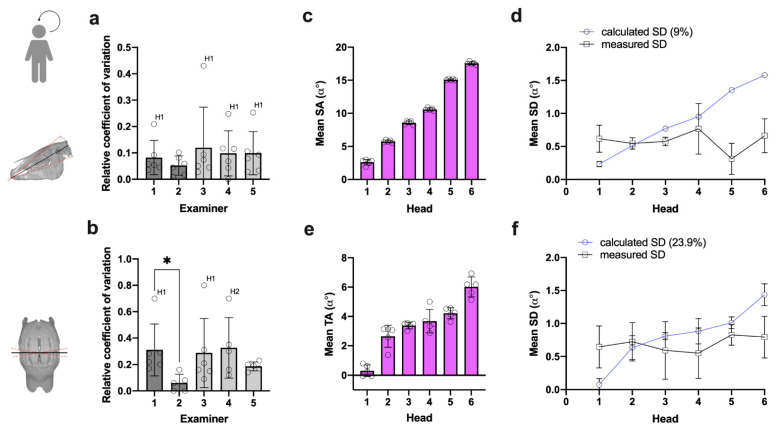
Analysis of SA and TA measurement repeatability. (**a**,**b**) Mean relative SA and TA COV per examiner (dots = mean for individual heads); (**a**) SA: *n* = 16(15) measures per head; Kruskal–Wallis test, *p* = 0.837. (**b**) TA: *n* = 16(13, head 1) measures per head; Kruskal–Wallis test, *p* = 0.025; Dunn’s multiple comparisons, * *p* = 0.021. (**c**,**e**) Heads ranked after mean SA magnitude (dots = mean for individual examiner) to analyze (**d**,**f**) scale-level independency of measurement dispersion; (**d**) SA: *n* = 16(15) measurements per head*examiner; linear regression analysis, calculated SD: *p* < 0.0001 (*R^2^ =* 0.990), measured SD: *p* = 0.766 (*R^2^ =* 0.003). (**f**) TA: *n* = 16(15) measurements per head*examiner; linear regression analysis, calculated SD: *p* < 0.0001 (*R^2^ =* 0.832), measured SD: *p* = 0.403 (*R^2^ =* 0.034).

The mean relative COV was 0.09 for SA and 0.239 for TA across all examiners and heads, implying an average 9% and 23.9% dispersion of individual measures around the mean, respectively (Figure 6a,b). At the given SA scale level of α 0–18° (MIN–MAX all heads), this represents a mean SD of α 0.58° (±0.13°) for repeated SA measurements across all heads and examiners (Table 2), indicating clinically acceptable SA measurement repeatability. The mean SD of α 0.69° (±0.24°) for repeated TA measurements (Table 2) is similarly acceptable but produced higher COVs, which was due to a generally lower scale level of α -3–8° (MIN–MAX all heads).

Although SA COV (except for examiner 2) was highest in head 1 (Figure 6a), the absolute scatter of repeated measures was similar to other heads when considering the low scale-level of mean SA α 2.4° in this head (Figure 6c). This also applies to head 1 and 2 in TA measures (Figure 6b,e).

To display consistency and scale-level independency of SA and TA measurement dispersion, we rearranged heads according to increasing angle values (Figure 6c,e) and opposed both the calculated SDs (9%, 23.9%) and measured SDs. Linear regression analysis revealed that the actual SD of repeated SA and TA measures stays largely the same irrespective of the head and head’s scale level (Figure 6d,f). These results prove that the mean dispersion of repeated SA and TA measures did not vary upon scale-level differences and are similar in different examiners. In contrast to subjective reports on difficulties identifying reference landmarks in TA measures on cadaver heads, no marked differences in individual examiners’ SA and TA measurement dispersion were detected comparing frozen-thawed heads and frozen-thawed vs. the fresh head.

The within examiner SA COVs did not decisively differ between examiners, although the mean COVs of experienced examiners were slightly lower (examiner 1 = 8.2% ± 6.5%; examiner 2 = 5.2% ± 3.6%) (Figure 6a). Examiner 2 (experienced) showed lower mean TA COVs (6.2% ± 6.8%) than the rest of the examiners (27.7% ± 10.2%). However, no systematic difference between experienced and inexperienced examiners was detected. Statistical comparison even showed the largest difference between the two experienced investigators (Figure 6b).

Based on the results, we assume that criteria for positive instrument validation for intraexaminer variability of SA and TA measures, using specified reference points, could be met.

### 3.4. Data Reproducibility Was Higher in SA Measures under Given Reference Landmarks

**Figure 7:**
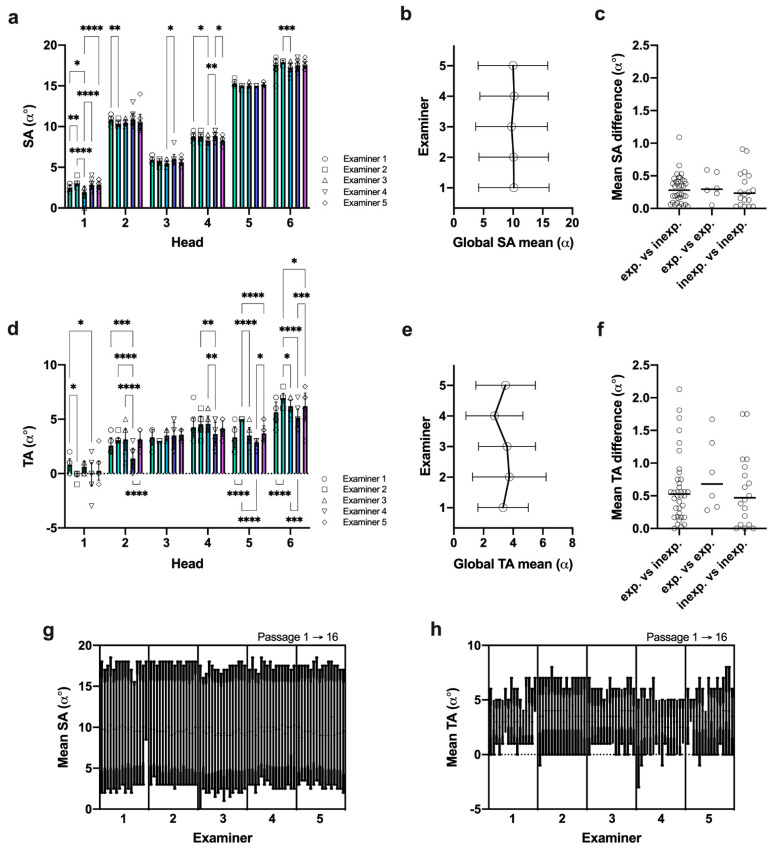
Analysis of SA and TA measurement reproducibility. (**a**,**d**) SA and TA measures per examiner*head (symbols = individual examiner measures); SA: *n* = 16(15), TA: *n* = 16(13, head 1) measures per head; two-way ANOVA, *p* < 0.0001; Tukey’s multiple comparisons, * *p* < 0.05, ** *p* < 0.01, *** *p* < 0.001, **** *p* < 0.0001. (**b**,**e**) Global SA and TA mean ± 95% confidence intervals. (**c**,**f**) Examiner dependency analysis for mean angle differences; exp. vs. inexp.: *n* = 36, exp. vs. exp.: *n* = 6, inexp. vs. inexp.: *n* = 18; Kruskal–Wallis test, *p* = 0.692 (SA), *p* = 0.630 (TA). (**g**,**h**) Nested angle values in passage chronology; SA: *n* = 16(15), TA: *n* = 16(13) measures per head.

We determined *MaPHorse1* interexaminer variability (reproducibility) comparing single examiner SA and TA measurements on different heads. Although statistical analyses showed that the measured SA values in some heads differed significantly for certain examiner combinations (Figure 7a), this can be considered clinically acceptable. The global difference between examiner means was negligible (Figure 7b), wherein the calculated mean difference of SA values ranged from α 0.12° ± 0.08° (head 5) to α 0.51° ± 0.35° (head 1) (Table 3). The highest calculated difference of mean SA values was α 1.09°, detected for examiner 2 vs. 3 in head 1 (α > 1° in 1/60 observations). Low spread of repeated SA measures is also indicated by a small interquartile range (IQR_Mean_: α 0.33°, IQR_MIN_: α 0.00°, IQR_MAX_: α 1.00°) and span (mean span: α 1.90° ± 1.10°) across different examiners and heads (Table S5). The observed measurement differences were distributed irrespective of the examiner’s level of experience (Figure 7c).

In the TA measures, more pronounced differences were observed between single examiners (Figure 7d). Ranging from α 0.26° ± 0.21° (head 3) to α 0.63° ± 0.72° (head 2), the calculated mean differences of mean TA values were slightly higher than for SA (Table 3) but at a much lower scale level. The highest difference of mean TA values was 2-fold higher (examiner 2 vs. 4 in head 5) compared to SA measures, and cases with α > 1° occurred 12 times more often (12/60 observations). The overall higher difference becomes more obvious looking at global examiner means (Figure 7e). Despite mean differences comparable to the SA, the spread of repeated TA measures was higher, exhibiting an average 2.2-fold higher interquartile range (IQR_Mean_: α 0.73°, IQR_MIN_: α 0.00°, IQR_MAX_: α 2.25°) and higher span (mean span: α 2.33° ± 1.30°) across different examiners and heads (Table S5). In the TA measures, the range of values within each examiner was between α 0° and α 5°, with a large proportion ≥α 3° (≤α 1° = 26.7%; >α 1° = 73.3%; ≥α 3° = 43.3%). Similar to SA, the mean TA measurement differences were not influenced by examiners’ level of experience (Figure 7f).

Nested display of SA and TA values revealed that SA measures were much more consistently reproduced by individual examiners over the course of different passages (Figure 7g,h).

The results indicate high measurement reproducibility for SA measures using proposed bony TMJ landmarks and suggest studying other anatomical landmarks for TA measures to lower data scatter and improve clinical acceptability.

### 3.5. Inexperienced Examiners Rapidly Adapt to the Measurement Procedure

**Figure 8:**
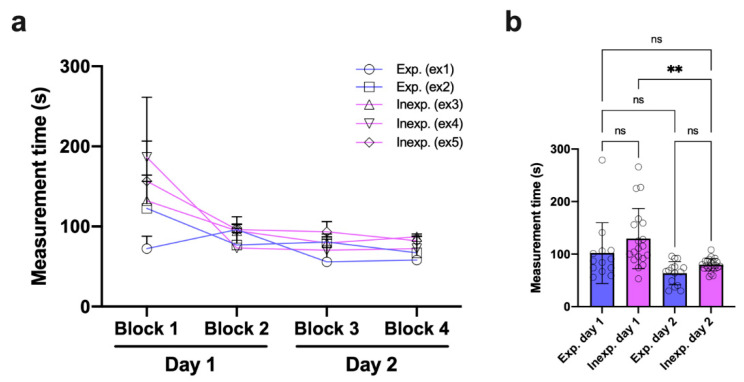
Measurement time analysis. (**a**) Required time separated by examiner and measurement block. (**b**) Required time comparing experienced and inexperienced examiners on experiment days 1 and 2; Kruskal–Wallis test, *p* < 0.0001; Dunn’s multiple comparisons, ** *p* = 0.004; Day 1: exp. (*n* = 13), inexp. (*n* = 19); Day 2: exp. (*n* = 15), inexp. (*n* = 22). ns, non-significance.

Time measurements were used to gather the average duration for orthodontic measurements comparing experienced and inexperienced examiners. The time required for individual orthodontic measurements was higher at the beginning of the experiment (block 1), whereas experienced examiners performed faster (Figure 8a). Both experienced examiners already had extensive routines in terms of using *MaPHorse1* before the experiment in >5000 horses during dental prophylaxis. In the following measurement blocks 2–4, the time required decreased, with the times of experienced and inexperienced examiners converging (Figure 8a). The values were already comparable after 24/96 repetitions. All the time measurements are listed in Table S6.

The overall time required for measurements did not systematically differ between experienced and inexperienced examiners on either day of the experiment. However, the calculated difference was slightly higher on day 1. Inexperienced examiners improved their performance, requiring markedly less time for measurements on day 2 of the experiment (Figure 8b).

## 4. Discussion

Standardized static and dynamic orthodontic measurements during routine dental prophylaxis can provide essential population data for more accurate diagnostics and effective treatment planning but also evaluation of treatment success, detailed patient follow-up, and client communication. In equine dentistry, it is common to subjectively appraise occlusal angles and the need for odontoplasty, as well as treatment outcomes, without applying objective metric analyses. We, therefore, conducted this blinded block-randomized cadaver study evaluating intra- and interexaminer variability in repeated SA and TA measures using the orthodontic gauge device *MaPHorse1*. The results indicate scale-level-independent high repeatability for both SA and TA measures. Referring to bony TMJ landmarks, the SA measures also revealed high reproducibility. Despite high methodical repeatability, the TA measures shared lower reproducibility using soft tissue landmarks. The general measurement performance was not substantially impacted by the previous experience of the examiners.

Gathering reliable population data on any dental or dentofacial angles in the horse requires validation of orthodontic measuring methods, which is currently largely missing. For validation of clinical measures, intra- and interexaminer variability are key parameters to assess measurement repeatability and reproducibility, respectively [33]. Both are widely used parameters in human orthodontics [34,35], yet just one previous study reported intraexaminer variability testing in computed tomography-based single-examiner cephalometrics to assess interincisal angulation in the horse [13]. Use of standardized, e.g., deep learning, algorithms to perform automated landmarking and measures in imaging-based cephalometrics may not require examiner variability testing but does necessitate other proof in terms of measuring accuracy and reliability and also comparison between different solutions [36]. Examiner variability analysis should be imperative when orthodontic measures are performed manually or semiautomated by different examiners at different skill levels and when data comparability is pursued. Our study, for the first time, presents data on examiner variability using *MaPHorse1*, a clinical gauge device that can be used by equine dental physicians to measure incisor occlusal surface angles. Although the results show examiner variability largely in the sub-angular range, this study does not consider other measuring methods, is confined to one anatomical landmark per measured angle, and is limited to cadaver specimens. Thus, affirmation of method performance requires further methods and landmarks to be validated, in vivo investigations, and subsequent data comparison. Even though experienced study participants reported parts of angle measurements being more easily performed in live horses, standards for head positioning and used landmarks have yet to be established.

Despite missing SA and TA population data from validated measures, incisor shortening and occlusal surface alignment are recommended in horses to restore functional occlusion after cheek tooth equilibration or dependent from incisor occlusal surface abnormalities [8,16,17]. Normal age-related morphometric changes in the incisors are a known cause of periodontopathy-promoting biomechanical stress distribution patterns in the periodontium of horses [37], which could be further promoted by treatment-related unphysiological alignment of occlusal surface angles. Measurements based on validated clinical gauge devices, for which our study attempts to pave the way, or validated imaging-based cephalometric protocols may help in obtaining sufficient data to unravel individual treatment needs.

The investigated rostro-caudal inclination of the incisor table is referred to as the SA [2]. Various reference points and lines are described to determine this angle’s normocclusal state in the horse. Accordingly, it is assumed that, with normocclusion, the extended incisor occlusal surface plane should cross the eye or ear ground [9,10], the TMJ level [26], or should be α 5° above the TMJ level [20]. Others specify SA normocclusion when the occlusal surface plane aligns parallel to the facial crest [8] or is inclined α 10–15° to a reference plane determined by the inferior interalveolar margins [7]. Figure S1 visually highlights these heterogeneous assumptions and substantiates the lack of evidence-based consensus regarding a normal healthy SA range, malocclusal state, and, e.g., age-related angle dynamics or dependence on other covariate factors. Listmann et al. (2017), in their CT-based 3D cephalometric study on incisor SA and TA, referred to the previously described facial crest and superior interalveolar margin as inappropriate reference lines due to their curved morphological shape, and they chose internal anatomical points to define a median (midsagittal) and transversal reference plane. They also tested the inferior interalveolar margin, which, due to its straight appearance, resulted in a more reliable and clinically applicable reference line [2]. However, with the device investigated here, no internal landmarks can be used and the inferior interalveolar margins appeared not applicable considering device buildup. We chose superficial bony TMJ landmarks to bilaterally assess SAs, assuming age-related morphometrical changes of other maxillofacial structures, as already investigated and shown in horses up to 12 months of age [38], to play a certain role also in older individuals. In humans, craniofacial skeletal morphometry changes throughout life, e.g., by reduction in facial height or increase in facial width and depth [39,40]. Accordingly, age-related changes can impact consistency of reference structures in longitudinal studies, as was yet shown in human cephalometrics [41]. The TMJs are central biomechanical subunits to the masticatory process representing the center of rotational and translational jaw movements [42]. In human cephalometrics, reference points related to the TMJs are presently used for angle measures [43,44]. In horses, the direct biomechanical association of the TMJs and incisor arcades at incisor landing/occlusion [5] assumingly also makes them a suitable reference unit.

The investigated latero-lateral inclination of the incisor table is referred to as the TA [2]. Literature reports a normal TA with the occlusal table inclined perpendicular (α 90°) to a median or paramedian/sagittal plane [7,8,19]. Others assume normocclusion when antagonistic incisors meet in a plane aligning parallel to the ground [4,19]. With the latter assumption, it must be noted that use of reference structures that align with a coordinate system external to the object of interest could easily lead to angle misinterpretation, e.g., due to tilted head position (Figure S1b). The clinical measurement method investigated here restricts to the use of surface reference points on the investigated object. The manufacturer recommends alignment of the frontal bar to the left and right bulbus to measure TA in live horses [20], but we observed enophthalmos and slight lateral canthal distortion using frozen-thawed cadaver heads. In human medicine, it is well described that the eyes show a distinct tendency to postmortem dehydration and decreased intraocular pressure [45]. Despite changes in the bulbi and position of lateral canthi, the medial canthi of the eyes represented reliable reference points as there was no significant change in soft tissue position. This could be attributed to the medial canthal ligament firmly connecting the respective medial corner of the eye to the periorbital fascia and orbital rim [46]. We, therefore, decided to align the frontal bar parallel to the left and right medial canthus.

Schlicher et al. (2012), in their study on precision and consistency of cephalometric landmark identification, opine that error distribution for most landmarks reflects the difficulty in locating the landmarks [47]. Although examiners reported easier bony landmark identification in the TMJ region compared to soft tissue landmarks in the eye region, measurement repeatability was comparably low in both SA and TA measures. However, there was slightly higher variability and less passage consistency in TA measures at an interexaminer level. This may be attributed to individual examiners perceiving eye soft tissue landmarks so that repeated measurements do not scatter much, but landmarks are not necessarily located and used in the same way by other examiners. Interexaminer variability was comparable in frozen-thawed cadaver heads and the fresh one; thus, cadaver quality may not have been the main reason for examiner differences. We suggest that palpatory guidance of the lateral bar to align with bony TMJ landmarks led to higher data reproducibility and consistency of SA measures. According to subjective examiner reports and obtained data, it appeared more difficult aligning the frontal bar parallel to a virtual line connecting the medial canthi with the naked eye. To improve TA measurement reproducibility, a modified approach that utilizes the same bony TMJ landmarks as in SA measures could be used. With this approach, the TA is gathered referring to a TMJ reference plane and corrected for possible influence of oculofacial asymmetries that can impair TA reads when using ocular reference points to describe masticatory interrelations. The approach again includes a TA measurement using ocular reference structures and double-sided SA measurement amended by a subsequent TA correction if SA differences occur left versus right (Figure S2; Video S2). Despite overall non-statistically significant left versus right difference that, on average, was in the subangular range, in 48% of measurements, a correctable difference occurred with α 1° in 97% and α up to 5° in 3% of cases. TA correction via TMJ landmarks may have led to an approximation of measurement reproducibility to the level determined for the SA. However, this approach needs validation as well.

Our study did not primarily intend on delivering population data on either angle. However, mean SAs (left, right) deviated α 0–18° in reference to bony TMJ landmarks, which is similar to previous studies. Pellachin (2013) described SAs ranging between α -5° (just ventral to TMJ level) and 20° in most of the 2700 examined cases using *MaPHorse1* and equal landmarks. The vast majority of cases (>90%) showed SAs above the TMJ level [20]. Listmann et al. (2017) also described SAs being at or above the TMJ level in 95% of cases [2]. The mean TA (± SD) was α 3.4° ± 1.9°, which is comparable to the mean angle range of α 3.5–6.8° previously observed [2]. Since we and others have measured a wide range of angles, it is reasonable to assume that there is a certain normocclusal angle range, and possibly also differences between different breeds and age groups. However, it should be noted that, compared to CT-based single tooth measurements in the study by Listmann et al. (2017), only one global angle value can be determined with the investigated device.

Landmark identification errors are considered a major source of error in cephalometric studies [47]. Training of examiners aims at reducing landmark identification errors, which further maintains interexaminer variation at a clinically acceptable level [48]. In our study, there was no systematic difference between experienced and inexperienced examiners in measuring either of the angles. The time required to carry out measurements plainly differed in block 1/4, with inexperienced examiners requiring more time, but rapidly approached the level of experienced examiners in block 2/4. Considering the findings on SA and TA measurement variability with *MaPHorse1*, the results suggest that even inexperienced examiners can perform rapid and valid clinical orthodontic measurements without requiring much training.

## 5. Conclusions

The investigated device could help deciphering incisor table angle normocclusal range and angle dynamics and may help to determine individual angle correction need and treatment success at high repeatability and reproducibility levels. In this cadaver study, the SA and TA measures were equally repeatable, but, compared to the high reproducibility of the SA measures using bony TMJ reference landmarks, the TA measures using soft tissue landmarks of the eye region performed slightly inferiorly at the interexaminer level. With a proposed procedural amendment, TA measures may also perform with higher reproducibility and consistency. Since the general measurement performance was not substantially impacted by the previous experience of the examiners, this device can be easily implemented into clinical practice.

## Figures and Tables

**Table 1:** Animal data and condition of heads used for orthodontic measurements.

Horse No.	Breed	Sex	Age	Coat	Condition
1	Riding Pony	mare	22	black	frozen-thawed
2	WB ^1^	gelding	21	brown	frozen-thawed
3	WB	gelding	8	brown	frozen-thawed
4	WB	mare	12	brown	frozen-thawed
5	WB	mare	13	black	frozen-thawed
6	WB	mare	12	brown	fresh

^1^ WB, warmblood.

**Table 2:** Repeated SA and TA measures SDs (α°) for individual examiner and head combinations.

Examiner	ITA	Head 1	Head 2	Head 3	Head 4	Head 5	Head 6	Mean SD
1	SA	0.52°	0.49°	0.55°	0.50°	0.53°	0.95°	0.59°
	TA	0.56°	0.73°	0.72°	0.86°	0.98°	0.96°	0.80°
2	SA	0.31°	0.46°	0.46°	0.52°	0.13°	0.30°	0.36°
	TA	0.25°	0.25°	0.00°	0.74°	0.00°	0.44°	0.28°
3	SA	0.83°	0.52°	0.51°	0.61°	0.40°	0.79°	0.61°
	TA	0.50°	0.96°	0.52°	0.73°	0.73°	0.54°	0.66°
4	SA	0.71°	1.25°	0.69°	0.60°	0.00°	0.76°	0.67°
	TA	1.06°	0.96°	1.21°	1.09°	0.34°	0.81°	0.91°
5	SA	0.72°	1.12°	0.52°	0.65°	0.50°	0.51°	0.67°
	TA	0.86°	0.72°	0.51°	0.72°	0.70°	1.22°	0.79°
**Mean**	**SA**	**0.62°**	**0.77°**	**0.55°**	**0.58°**	**0.31°**	**0.66°**	**0.58°**
	TA	0.65°	0.72°	0.59°	0.83°	0.55°	0.79°	0.69°

ITA, incisor table angle; SA, sagittal angle; TA, transversal angle; SD, standard deviation; n = 16(15) per examiner*head*angle.

**Table 3:** Mean SA and TA difference (α°) between examiners for different heads.

Examiner Combination	ITA	Head 1	Head 2	Head 3	Head 4	Head 5	Head 6	Mean
Ex 1 vs. Ex 2	SA	0.56°	0.59°	0.28°	0.05°	0.23°	0.31°	0.34°
	TA	0.86°	0.50°	0.33°	0.28°	1.67°	1.31°	0.83°
Ex 1 vs. Ex 3	SA	0.53°	0.41°	0.46°	0.53°	0.17°	0.34°	0.41°
	TA	0.18°	0.56°	0.17°	0.31°	0.17°	0.56°	0.32°
Ex 1 vs. Ex 4	SA	0.35°	0.16°	0.07°	0.03°	0.20°	0.09°	0.15°
	TA	0.86°	1.19°	0.17°	0.63°	0.46°	0.50°	0.63°
Ex 1 vs. Ex 5	SA	0.38°	0.38°	0.34°	0.47°	0.04°	0.06°	0.28°
	TA	0.55°	0.56°	0.23°	0.13°	0.35°	0.56°	0.40°
Ex 2 vs. Ex 3	SA	1.09°	0.19°	0.19°	0.48°	0.06°	0.66°	0.44°
	TA	0.69°	0.06°	0.50°	0.03°	1.50°	0.75°	0.59°
Ex 2 vs. Ex 4	SA	0.22°	0.44°	0.34°	0.08°	0.03°	0.41°	0.25°
	TA	0.00°	1.69°	0.50°	0.91°	2.13°	1.81°	1.17°
Ex 2 vs. Ex 5	SA	0.19°	0.22°	0.06°	0.42°	0.19°	0.38°	0.24°
	TA	0.31°	0.06°	0.56°	0.41°	1.31°	0.75°	0.57°
Ex 3 vs. Ex 4	SA	0.88°	0.25°	0.53°	0.56°	0.03°	0.25°	0.42°
	TA	0.69°	1.75°	0.00°	0.94°	0.63°	1.06°	0.84°
Ex 3 vs. Ex 5	SA	0.91°	0.03°	0.13°	0.06°	0.13°	0.28°	0.26°
	TA	0.38°	0.00°	0.06°	0.44°	0.19°	0.00°	0.18°
Ex 4 vs. Ex 5	SA	0.03°	0.22°	0.41°	0.50°	0.16°	0.03°	0.22°
	TA	0.31°	1.75°	0.06°	0.50°	0.81°	1.06°	0.75°
**Mean**	**SA**	**0.51°**	**0.29°**	**0.28°**	**0.32°**	**0.12°**	**0.28°**	**0.30°**
	**TA**	**0.42°**	**0.63°**	**0.26°**	**0.51°**	**0.57°**	**0.59°**	**0.64°**

ITA, incisor table angle; SA, sagittal angle; TA, transversal angle; *n* = 16(15) measures per examiner*head. Colored values indicate mean angle differences α > 1°.

## Data Availability

Data are contained within the article or Supplementary Materials.

## Supplementary Materials

The following supporting information can be downloaded at: https://www.mdpi.com/article/10.3390/vetsci9090481/s1, Table S1: Block-randomization list; Table S2: Examination protocol; Table S3: SA data; Table S4: TA data; Table S5: SA and TA data spread; Table S6: Measuring duration data; Figure S1: Heterogeneity of assumed SA normocclusion and description of TA reference frame problem; Figure S2: Process flow chart for double-sided SA reading and TA correction procedure; Video S1: 3D model displaying procedure of TA and exemplary right-sided SA reading; Video S2: Double-sided SA reading and TA correction procedure. References [49] are cited in the supplementary materials.
