# Supplementary material for: Intra- and Interexaminer Measurement Variability Analysis of an Orthodontic Gauge Device to Determine Incisor Occlusal Surface Angles in the Horse
Source: Vet Sci. 2022 Sep 7;9(9):481. doi: 10.3390/vetsci9090481 (PMC9506125; doi:10.3390/vetsci9090481)
Supplement: Supplementary file 1 [file vetsci-09-00481-s001.zip › Figure S1.pdf]

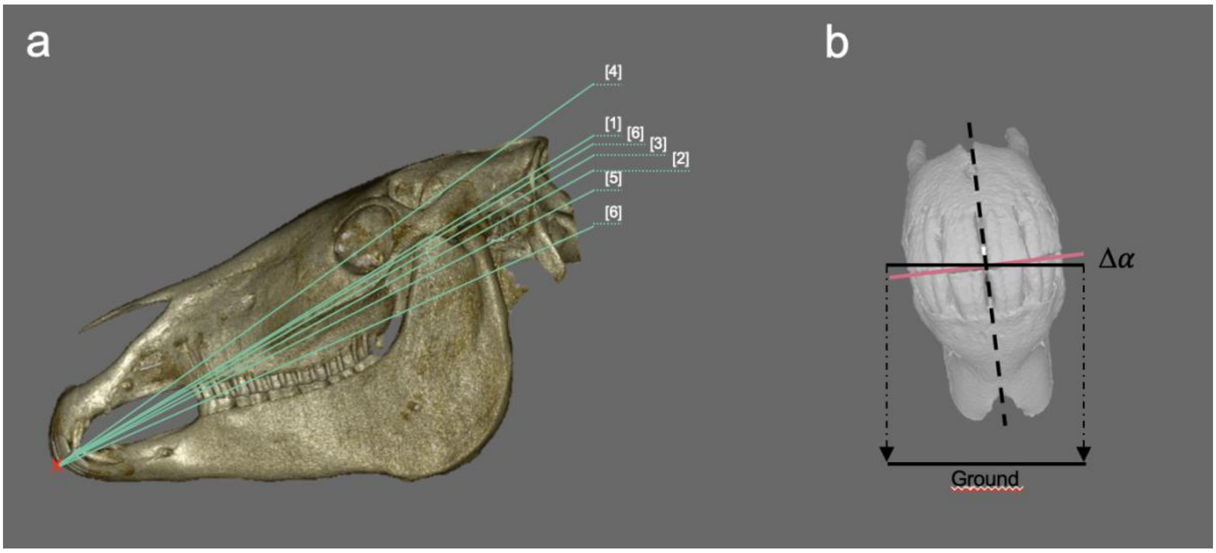

Figure S1. Heterogeneity of assumed normocclusion inclination of incisor occlusal surface SA (a) and visualization of the problem interpreting TA using an external reference frame at tilted head position (b).

References:

[1] D. O. Klugh, "Anatomical characteristics of equine dentition," in *Principles of Equine Dentistry*, 1st ed., D. O. Klugh, Ed. London: Manson Publishing, 2010, pp. 27–48.

[2] D. O. Klugh, "Principles of occlusal equilibration," in *Principles of Equine Dentistry*, 1st ed., D. O. Klugh, Ed. London: Manson Publishing, 2010, pp. 67–78.

[3] K. Ros, "Biomechanics," in *Textbook of Equine Dentistry*, 1st ed., C. Vogt, Ed. Stuttgart: Schattauer GmbH, 2011, pp. 31–48.

[4] M. Pellachin, "Objective Measurements of occlusal angles," in *Proceedings of the 11th IGFP Conference*, 2013, pp. 45–57.

[5] B. A. Rucker, "Incisor and molar occlusion: normal ranges and indications for incisor reduction.," in *Proceedings of the 50th Annual Convention of the American Association of Equine Practitioners, Denver, Colorado, USA*, 2004, pp. 7–12.

[6] T. Allen, "Examination," in *Manual of Equine Dentistry*, 1st ed., T. Allen, Ed. St. Louis: Muleicorn Press, 2008, pp. 67–88.
