# Supplementary material for: Intra- and Interexaminer Measurement Variability Analysis of an Orthodontic Gauge Device to Determine Incisor Occlusal Surface Angles in the Horse
Source: Vet Sci. 2022 Sep 7;9(9):481. doi: 10.3390/vetsci9090481 (PMC9506125; doi:10.3390/vetsci9090481)
Supplement: Supplementary file 1 [file vetsci-09-00481-s001.zip › Figure S2.pdf]

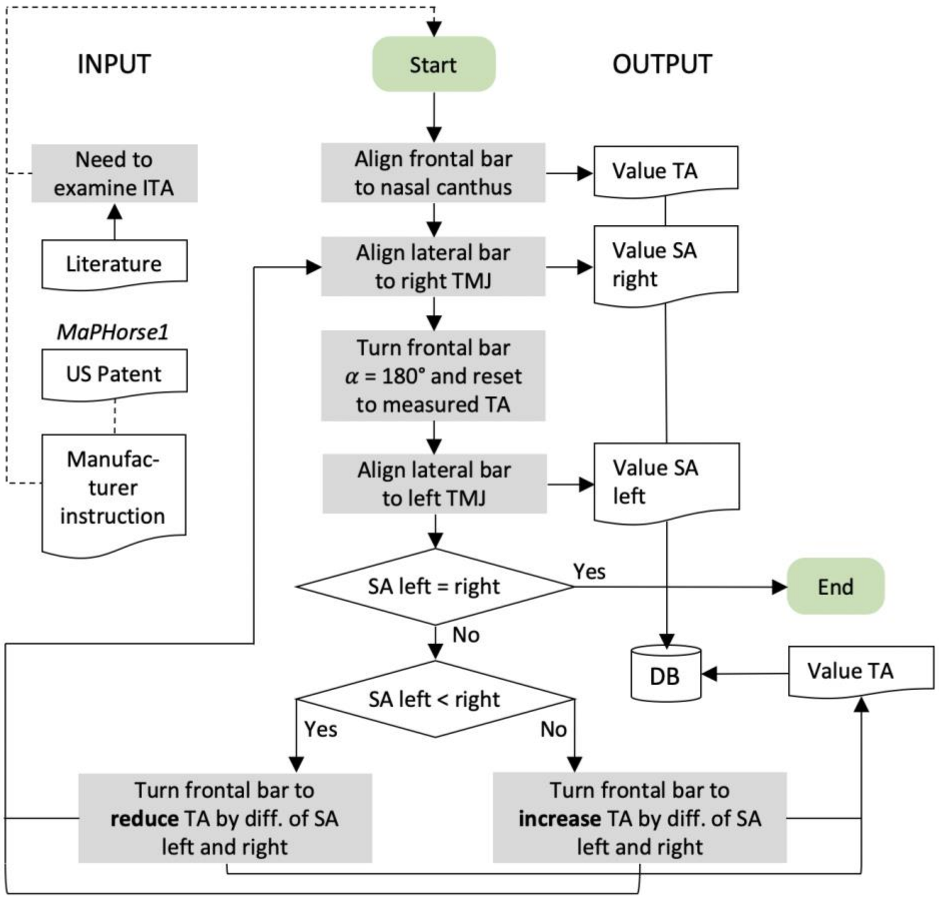

Figure S2. Process flow chart of corrective double sided reading for TA measures using TMJ bony landmarks.
