# Supplementary material for: Intra- and Interexaminer Measurement Variability Analysis of an Orthodontic Gauge Device to Determine Incisor Occlusal Surface Angles in the Horse
Source: Vet Sci. 2022 Sep 7;9(9):481. doi: 10.3390/vetsci9090481 (PMC9506125; doi:10.3390/vetsci9090481)
Supplement: Supplementary file 1 [file vetsci-09-00481-s001.zip › Table S1.pdf]

Table S1. Block-randomization list.

Block	Passage		Box 1	Box 2	Box 3	Box 4	Box 5	Box 6
1	1	**Head number**	1	2	3	4	5	6
	2		2	6	4	3	1	5
	3		4	6	2	1	5	3
	4		1	5	2	4	3	6
2	1		6	4	1	2	3	5
	2		5	3	1	6	2	4
	3		3	1	5	4	6	2
	4		4	1	6	5	2	3
3	1		6	5	3	4	2	1
	2		4	6	1	2	5	3
	3		5	3	2	6	1	4
	4		3	1	5	4	2	6
4	1		2	6	4	3	5	1
	2		4	3	6	1	2	5
	3		1	2	3	5	4	6
	4		6	4	5	2	1	3

Fixed factors: block, passage, and box; random range: head 1–6.
