# Supplementary material for: Intra- and Interexaminer Measurement Variability Analysis of an Orthodontic Gauge Device to Determine Incisor Occlusal Surface Angles in the Horse
Source: Vet Sci. 2022 Sep 7;9(9):481. doi: 10.3390/vetsci9090481 (PMC9506125; doi:10.3390/vetsci9090481)
Supplement: Supplementary file 1 [file vetsci-09-00481-s001.zip › Table S2.pdf]

Table S2. Exemplary examination protocol for block 1 and observer 1.

Block	Passage	Observer	Measure	Box	TA (α)			SA (α) right	SA (α) left
					0°	3	4		
1	1	1	1	2					
			2	6					
			3	4					
			4	1					
			5	3					
			6	5					
1	2	1	1	4					
			2	6					
			3	3					
			4	5					
			5	2					
			6	1					
1	3	1	1	2					
			2	3					
			3	4					
			4	6					
			5	1					
			6	5					
1	4	1	1	2					
			2	5					
			3	6					
			4	1					
			5	3					
			6	4					

TA, transversal angle (3 = DGL-3, 4 = DGL-4); SA, sagittal angle.
